# Changes in Phenolic Compounds, Antioxidant Capacity, Color, and Microstructure of Hass Avocado (*Persea americana*) Peel During Ripening

**DOI:** 10.3390/molecules31183280

**Published:** 2026-09-16

**Authors:** Madeleine Perucini-Avendaño, Lorraine Salgado-León, Alberto Peña-Barrientos, Vanessa Sánchez-Quezada, Rocio Campos-Vega, Guadalupe Loarca-Piña

**Affiliations:** 1 Programa de Investigación y Posgrado en Ciencia de los Alimentos, Facultad de Química, Universidad Autónoma de Querétaro, Cerro de las Campanas S/N, Col. Centro, Santiago de Querétaro CP 76010, Mexico; lsalgado9406@gmail.com (L.S.-L.); vanelilla@hotmail.com (V.S.-Q.); chio_cve@yahoo.com.mx (R.C.-V.); 2 Centro de Nanociencias y Micro y Nanotecnologías, Instituto Politécnico Nacional, Unidad Profesional Adolfo López Mateos, Av. Luis Enrique Erro S/N, Gustavo a Madero, Mexico City CP 07738, Mexico; alberto_881025@hotmail.com

**Keywords:** *Persea americana*, nutraceutical composition, physicochemical properties, structure, microscopy techniques

## Abstract

Avocado peel is a high-value by-product rich in bioactive compounds; however, its structural and chemical integrity undergoes critical transitions during postharvest ripening. This study evaluated the influence of ripening stage (unripe, ripe, and overripe) on the chemical composition, color attributes, antioxidant capacity, and microstructural evolution of Hass avocado peel. Significant differences (*p* < 0.05) were observed in proximate composition and color coordinates among ripening stages. Overripe peel exhibited the highest concentration of total phenolic compounds (168.02 ± 3.28 mg GAE/g) and antioxidant capacity, determined by ABTS•+ (410.25 ± 1.01 µM TE/g) and FRAP (782.84 ± 2.62 µM TE/g). Environmental scanning electron microscopy (ESEM) and confocal laser scanning microscopy (CLSM) revealed progressive degradation of cuticular layers and chlorophyll-containing cells during ripening. Surface fractal dimension texture (FDT) significantly decreased from 1.54 ± 0.02 in unripe peel to 1.47 ± 0.01 in overripe peel, indicating a reduction in surface structural complexity. Principal component analysis (PCA) explained 99.0% of the total variance and revealed a clear association between FDT, chlorophyll fluorescence (λex = 450 nm), and color parameters, while total phenolic content and antioxidant capacity were associated with advanced ripening stages. These findings demonstrate that FDT is a promising indicator of peel senescence and suggest that structural disassembly during ripening is closely associated with enhanced phenolic extractability and antioxidant potential. This work provides a comprehensive framework for monitoring postharvest quality and supports the valorization of avocado peel as a source of bioactive compounds for food applications.

## 1. Introduction

*Persea americana* Mill. var. ‘Hass’ is a versatile food that can be consumed in different ways, mainly as fresh fruit. Mexico is the world’s leading producer and exporter, with an annual production of 2,529,581.41 tons [1]. The industrial production of avocado results in the generation of large amounts of waste, such as peel and seed, representing approximately 7–15% and 20–21% of the fresh weight of the fruit, respectively, thus increasing environmental pollution [2,3,4,5]. ‘Hass’ avocado is characterized as a climacteric fruit with an ovoid shape and skin color that changes from green at the immature stage to purple at maturity [6]. Its chemical composition is characterized by a high lipid content (12–24%), mainly composed of polyunsaturated and monounsaturated fatty acids, carbohydrates (8.64%), ash (1.66%), and moisture (72.3%) [7]. Ripening involves a series of coordinated metabolic reactions that affect morphology, biochemistry, physiology, and microstructure. These alterations modify quality attributes such as fruit composition, color, texture, and flavor, particularly in climacteric fruits such as avocado [8].

Microscopy and image analysis provide valuable tools for evaluating structural changes in plant tissues during ripening and relating them to physicochemical and biochemical properties [9]. In avocado, previous studies have characterized peel composition and some structural features [10]; however, the evolution of peel microstructure across distinct ripening stages and its relationship with antioxidant properties remains insufficiently understood. In particular, quantitative approaches capable of describing changes in surface structural complexity could complement conventional microscopic observations and provide an objective assessment of tissue organization during ripening. Fractal analysis provides a quantitative approach for describing the complexity and irregularity of biological structures and may therefore complement ESEM and CLSM observations of avocado peel. However, to our knowledge, the variation in surface fractal dimension texture (FDT) of Hass avocado peel throughout ripening and its association with color, chlorophyll fluorescence, total phenolic content, and antioxidant capacity have not been simultaneously investigated. Addressing these relationships may provide a more integrated understanding of the structural and biochemical changes occurring in avocado peel during ripening. Therefore, the objective of this study was to characterize changes in the chemical composition, phenolic compounds, antioxidant capacity, and microstructure of Hass avocado peel during ripening and to elucidate the relationships between structural transformations and the chemical and antioxidant properties of this agro-industrial by-product through multivariate analysis.

## 2. Results and Discussion

### 2.1. Effect of Maturity Stage on the Chemical Composition and Color of Persea americana

The chemical composition (dry basis) of avocado peels at different ripening stages is presented in Table 1. Protein and ash contents did not differ significantly among ripening stages. Protein values were higher than those previously reported for the same cultivar (1.51–8.1%) [10,11,12], whereas ash content was within the reported range (2–3.57%) [7,13]. Variability in avocado peel composition may be influenced by maturity and preharvest factors, including climate and soil conditions [10,12,14,15,16]. No significant differences were found in lipid content between UAP and RAP. However, a decrease of 7.6–8.01% was observed in the OAP sample. These values are within the range reported by several authors (2.89–11.04%) [10,11,12]. This wide range of variation may be associated with complex formation involving carbohydrates, proteins, or phenolic compounds, as well as preharvest factors [12,17]. Ash results did not present significant differences among the different maturity stages. Several authors have reported similar values for mature avocado peel of the same variety (2–3.57%) [7,13]. These percentages are mainly attributed to the presence of minerals such as potassium, calcium, magnesium, and sodium [18]. Carbohydrate composition showed no significant differences between UAP and RAP. However, an increase of 1.68% was observed in the OAP sample. The same trend was observed in total dietary fiber content, which increased by 8.31% in the mature stage compared with the immature stage. These results are similar to those reported by Salmeron-Ruiz [19] (71.36%) and Colombo and Papetti [2] (62–73.3%) for carbohydrates, and Araujo et al. [7] (67.80%) for total dietary fiber. These differences may be associated with a decrease in lipid content. During the unripe stage, a fraction of polysaccharides contributes to the transport of metabolites for cutin synthesis. As ripening progresses, cutin is degraded, and the ester or ether bonds formed between cutin and the carboxyl or hydroxyl groups of these polysaccharides are broken, increasing their content [20].

Avocado peel color parameters of UAP, RAP, and OAP, as determined by lightness (L*), chromatic coordinates (a* and b*), color saturation or intensity (Chroma, C*), and Hue° values, are shown in Table 1. The L* values underwent significant changes (*p* < 0.05) during ripening. Aguiló-Aguayo et al. observed similar behavior in avocados of the same variety subjected to pulsed light treatments [21]. These changes are mainly attributed to enzymatic browning as a consequence of the activation of polyphenol oxidase, which catalyzes oxidation reactions. This process is promoted by the rupture of cell membranes, causing the loss of functional cellular compartmentalization. This phenomenon was confirmed by the ESEM images, where progressive loss of cell structure is observed as ripening advances, increasing enzyme–substrate contact and consequently tissue browning [22]. As for the a* values, they were −19.45 ± 0.38 (UAP), −0.19 ± 0.06 (RAP), and 1.98 ± 1.20 (OAP), showing significant differences that indicate a shift toward the red component of the chromatic diagram (L*, a*, b*). Likewise, the b* values showed a tendency toward darker coloration. The chroma (C*) values of 25.04 ± 1.37 (UAP), 10.16 ± 2.20 (RAP), and 6.29 ± 1.49 (OAP) decreased significantly, as did the Hue° values. This color transition from green to purple/black during ripening has been reported in different avocado varieties and genotypes [21,23]. The visual appearance of the fruits at the different ripening stages, together with their corresponding CIELAB color parameters, is presented in Figure S1, supporting the progressive color changes observed instrumentally. Color saturation and Hue° indicate that postharvest ripening may involve browning phenomena associated with the polymerization of phenolic compounds, changes in kaempferol concentration and condensed tannins, as well as the oxidation of lipid compounds, which correlates with the decrease in lipid content observed in the proximate chemical analysis [17,21]. These perceptible color changes could serve as the basis for the development of technologies based on color determination, image analysis, and microscopic techniques for the efficient identification of ripening stages in different avocado varieties.

### 2.2. Influence of Maturity Stage on Total Phenolic Content and Antioxidant Capacity

The highest TPC was observed in OAP (168.02 ± 3.28 mg GAE/g), representing increases of 8.5% and 48.05% compared with UAP and RAP, respectively. This increase may be associated with structural changes during ripening that enhance phenolic extractability and with changes in anthocyanins, such as cyanidin-3-*O*-glucoside, which has been reported as the predominant anthocyanin in Hass avocado peel [23,24,25]. The TPC values obtained were higher than those previously reported for the same cultivar [10,15,26,27,28], which may reflect differences in geographical origin, maturity, storage conditions, and extraction methodology [28,29,30,31]. Antioxidant capacity differed significantly among ripening stages (Table 1). UAP showed the highest DPPH value (943.81 ± 5.40 µM TE/g), whereas OAP exhibited the highest ABTS•+ (410.25 ± 1.01 µM TE/g) and FRAP (782.84 ± 2.62 µM TE/g) values. The higher TPC, ABTS•+, and FRAP values in OAP coincided with advanced structural deterioration and peel darkening, suggesting increased accessibility of phenolic compounds during ripening. These responses may also reflect synergistic contributions from anthocyanins, procyanidins, flavonoids, and hydroxycinnamic acids present in avocado peel [15,25,27,28].

### 2.3. Effect of Ripening State on Avocado Peel Microstructure

**Figure 1:**
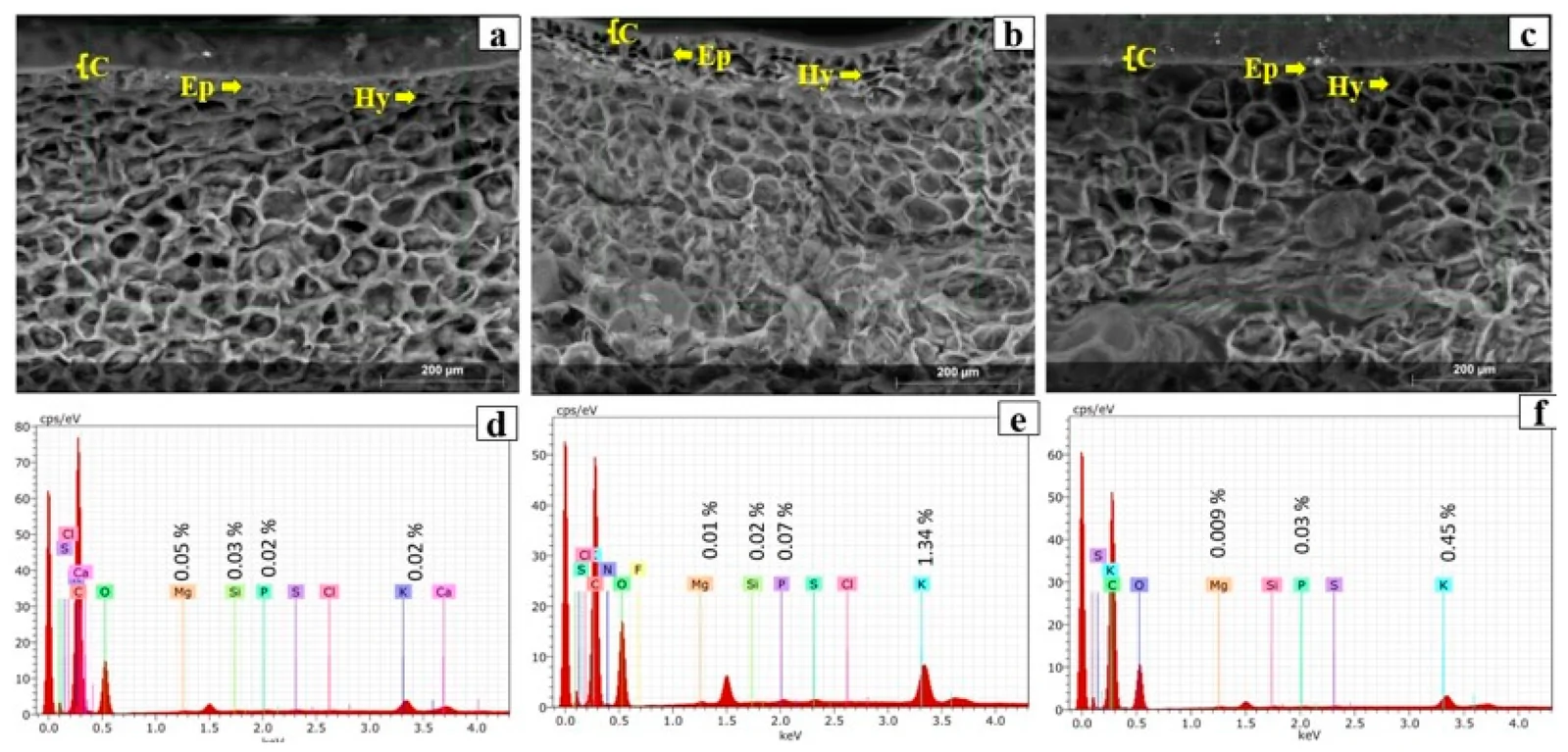
Images by ESEM (1) of (**a**) UAP, (**c**) RAP, (**e**) OAP at 200×, and their respective EDS spectrum (**b**,**d**,**f**). C: Cuticle, Ep: epidermis, Hy: hypodermis.

Structural changes in the peel were identified at the three maturity stages by ESEM and CLSM observations. Figure 1a,c,e shows the avocado peel morphology obtained by ESEM. The cuticle (C), epidermal cells (Ep), and hypodermal cells (Hy) can be clearly distinguished, consistent with previous descriptions of avocado peel anatomy [32,33]. During ripening, the avocado cuticle exhibited a significant (*p* < 0.05) increase in thickness from 11.86 ± 1.41 µm (UAP) to 15.94 ± 3.74 µm (RAP), followed by a marked decrease to 3.92 ± 0.99 µm in OAP. This reduction may be associated with increased peel surface tension caused by cell expansion and the synergistic action of cell wall-degrading enzymes, which are activated by the sharp increases in respiration and ethylene production that occur during fruit ripening [34].

The peel plays an important role in different fruits by regulating water absorption, gas exchange, protecting the fruit against biotic stress and mechanical injury, and maintaining fruit integrity [35,36]. Epidermal cells (Ep) exhibited an irregular quadrangular shape with intercellular spaces and are primarily associated with the cuticle, which plays a fundamental role in regulating water and gas exchange with the environment during fruit ripening and senescence. Beneath the epidermal layer are the hypodermal cells (Hy), which are isodiametric in shape, measure approximately 35–45 µm, and provide mechanical support to the peel.

Elemental microanalysis of the peel in all samples revealed the presence of minerals such as P, K, Mg, and Si (Figure 1b,d,f). Elemental analysis showed a slight decrease in the intensity of these minerals during ripening. These observations are consistent with the proximate chemical analysis, in which the ash content did not differ significantly among maturity stages and was mainly attributed to the presence of minerals such as potassium, calcium, magnesium, and sodium [18], findings that were corroborated by the EDS analysis. The elemental microanalysis (EDS) also revealed the presence of calcium (Ca) exclusively in unripe avocado peel (UAP) (Figure 1b), which correlated with the high structural integrity observed in the ESEM and CLSM images at this stage. In immature fruit tissues, Ca ions play a fundamental role in stabilizing the cell wall by forming ionic cross-links between the carboxyl groups of polygalacturonic acid chains through the well-known “egg-box” model of pectin gels [37,38]. The detection of Ca in UAP suggests the presence of a rigid pectin matrix that maintains cell-to-cell adhesion and limits cell wall extensibility.

Conversely, the absence of detectable Ca signals in RAP and OAP suggests the mobilization or leaching of these ions as ripening progresses. This process is typically driven by the increased activity of pectin methylesterase (PME) and polygalacturonase (PG), which solubilize the pectin fraction and disrupt Ca–pectate bridges [39]. The loss of this “ionic glue” explains the cellular collapse and thinning of the hypodermal (Hy) and epidermal (Ep) layers observed in the overripe samples, as the mechanical support provided by the Ca–pectin complexes is progressively compromised. The Ca percentage values were detected only in UAP. Previous studies have associated the presence of Ca with the carboxyl groups of cell wall pectin molecules, where Ca–pectin complexes contribute to tissue firmness and are progressively lost during fruit softening as ripening advances [31,38].

**Figure 2:**
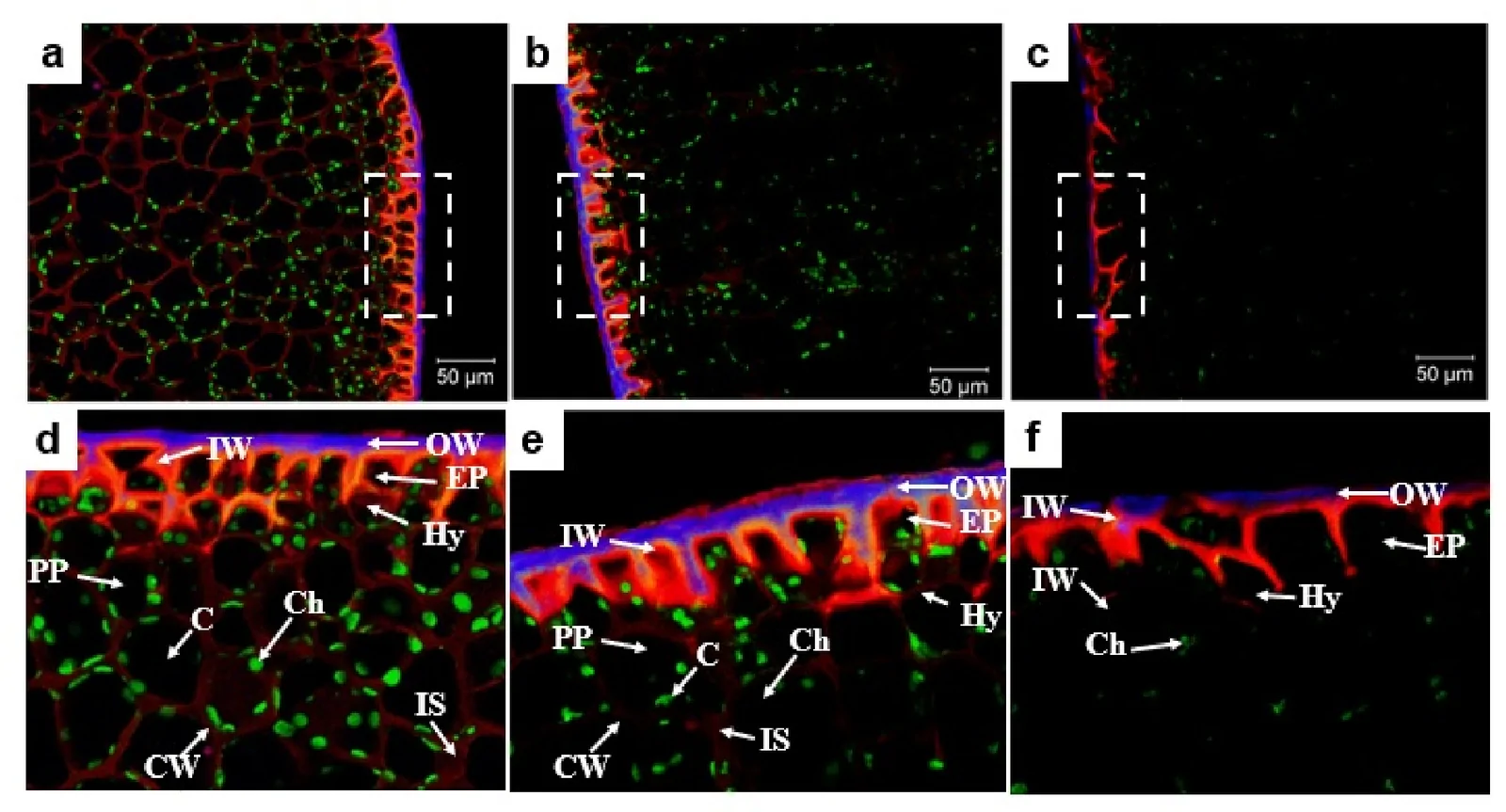
CLSM images of cross-sections of avocado peel at different ripening stages stained with Calcofluor White (purple), Rhodamine B (red), and autofluorescence (green). (**a**) UAP; (**b**) RAP; (**c**) OAP; (**d**–**f**) enlarged views of the regions indicated by the dashed boxes in (**a**–**c**), corresponding to UAP, RAP, and OAP, respectively. OW: outer wax layer; IW: inner wax layer; EP: epidermis; Hy: hypodermis; PP: pericarp parenchyma; IS: intercellular space; C: crystal; Ch: chloroplast; CW: cell wall.

Figure 2 shows the cross-sections stained with Calcofluor White (blue), Rhodamine B (red), and autofluorescence (green), observed by CLSM at the different ripening stages. The multilayer structure of the avocado peel was confirmed by CLSM, allowing clear visualization of the outer wax layer (OW), inner wax layer (IW), epidermis (Ep), hypodermis (Hy), and pericarp parenchyma (PP). The outer (OW) and inner (IW) cuticle layers exhibited higher fluorescence intensity in RAP, reaching a thickness of 8.91 ± 2.05 µm, and decreased markedly in OAP, consistent with the reduction in lipid content observed in the proximate chemical analysis. Although the multilayer structure was stained blue by Calcofluor White, a purple coloration was observed in the intermediate region due to the overlap between the fluorescence emitted by the upper layer, composed of cutin and epicuticular waxes, and the lower layer corresponding to the cell wall polysaccharides [40,41].

The extracellular membrane of the cuticle consists mainly of polymerized lipids, whose structure and composition vary according to plant species, organ type, and developmental stage [20,41]. Cutin and waxes are significantly reduced in mature fruits with the concomitant appearance of carotenoids, polyphenols, and polysaccharides, which may explain the decrease observed in the OAP samples. The outer wax (OW) layer exhibited a rough and irregular surface with numerous cracks of varying thicknesses, whereas the inner wax (IW) layer was more uniform in thickness and displayed fissures approximately along each radial wall of the epidermal cells. As observed, the cuticle is hydrophobic in nature because of its cutin and wax composition, protecting the cell wall of epidermal cells against dehydration [42,43]. The structure of the cuticle varies among plant species, organs, and developmental stages, ranging from a procuticle in emerging organs to a fully developed cuticle after tissue expansion has ceased [41,44]. Together, the cell wall and cuticle regulate peel permeability through their capacity to absorb or restrict water diffusion, depending on their structural organization and chemical composition [37,41].

The epidermis plays an important role in determining the rate of fruit expansion and providing mechanical support [45]. Changes in this tissue became evident as ripening progressed. Epidermal (Ep) cells exhibited a thickness of 16.83 ± 3.34 µm in UAP, followed by a slight decrease to 15.56 ± 1.42 µm in RAP, whereas marked thinning and disruption of these cells were observed in OAP. A similar behavior was observed for the hypodermal (Hy) cells, which are involved in radial and tangential cell division and contribute to fruit expansion. Schroeder described the development of a hypodermis consisting of one or two cell layers in ‘Hass’ avocado [32]. The innermost layer of the fruit peel is composed of parenchyma cells containing abundant chloroplasts. As ripening advanced, changes in the cell wall (CW) became evident, with an increase in the intercellular space, as shown in Figure 2a–c. This increase may be associated with a reduction in cell wall thickness and stiffness in avocado peel. UAP exhibited a thicker cell wall, whereas progressive ripening resulted in cell wall thinning, which may be related to the degradation of complexes formed by carbohydrates, lipids, and proteins.

**Figure 3:**
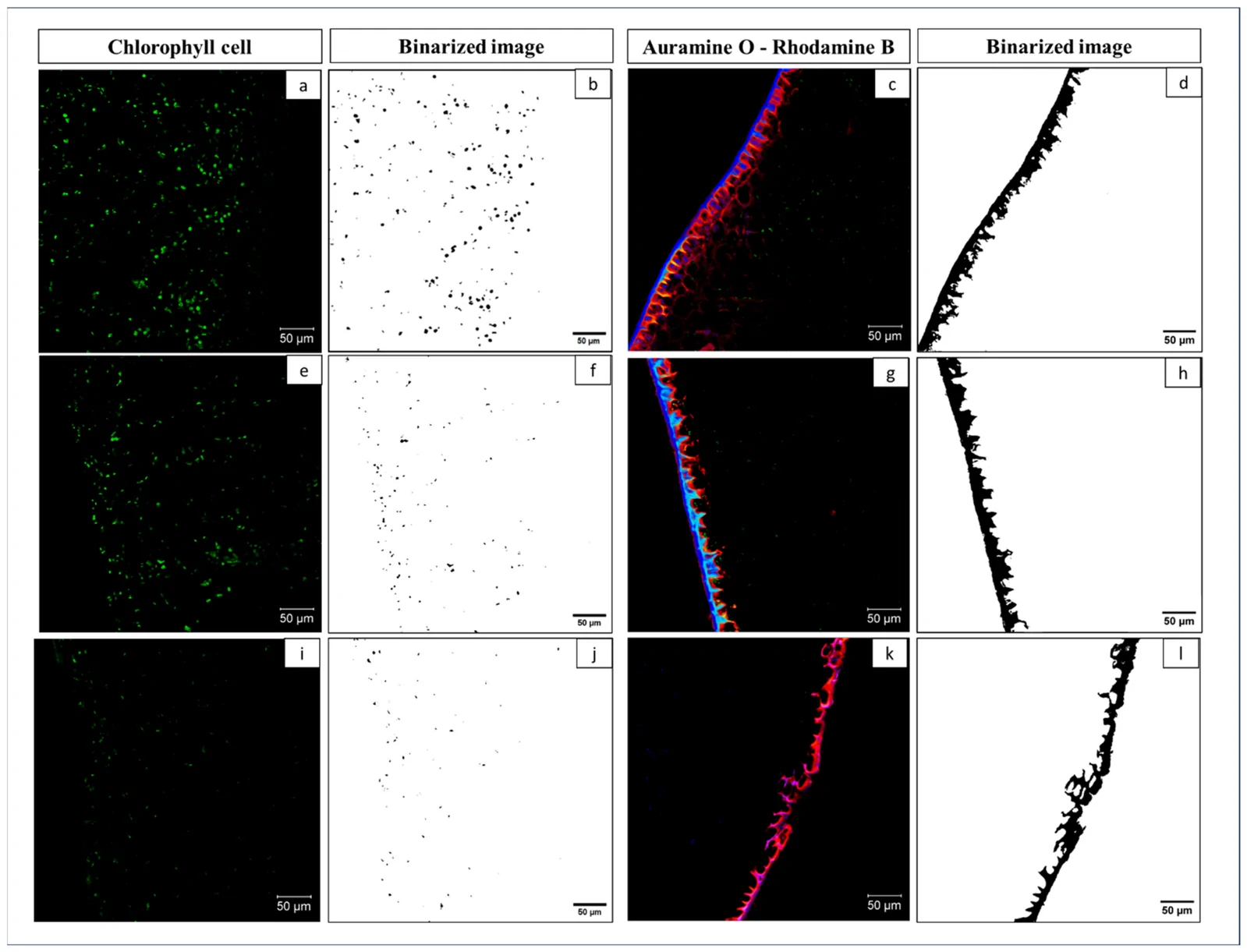
Images obtained by CLSM of a cross section of the samples. Image by autofluorescence (Chlorophyll cell) of UAP (**a**), RAP (**e**), OAP (**i**) and their respective binarized image by image analysis (**b**,**f**,**j**), staining with Auramine O–Rhodamine B (cuticle) UAP (**c**), RAP (**g**), OAP (**k**), and their respective binarized image (**d**,**h**,**l**).

Descriptive parameters of chlorophyll cell morphology and distribution were obtained from Figure 3a,e,i, and their values are listed in Table 2. The area, perimeter, and diameter values showed that chlorophyll cells exhibited a wide range of sizes, which decreased as ripening progressed, consistent with the autofluorescence emission intensities presented in Table 2. The aspect ratio and circularity parameters indicated that the cells displayed slightly elongated shapes. Regarding their distribution, chlorophyll cells were located within the parenchyma cell wall, as shown in Figure 2a.

The fluorescence intensity at λ450 nm is indicative of the presence of chlorophyll, which decreased dramatically from UAP to OAP, demonstrating extensive chlorophyll degradation during ripening. This loss of chlorophyll has been widely linked to the synthesis and accumulation of phenol-derived pigments and anthocyanins in the peel of avocados and other climacteric fruits [25]. In ‘Hass’ avocados, cyanidin-3-*O*-glucoside has been identified as the main anthocyanin in the peel at late maturity, coinciding with peel darkening and increased phenolic content [46].

Protein–chlorophyll complexes are associated with the thylakoid membrane, and their fluorescence is an indicator of the fluidity, stability, and organization of chloroplast membranes [47]. As avocado maturity increases, thylakoid destacking causes loss of physical integrity, photosynthetic capacity, and dissociation of pigment–protein complexes. This decrease in chlorophyll cell fluorescence correlates with Hue° and increased phenolic compounds (anthocyanin accumulation). The application of AI techniques facilitated and complemented the morphological study of chlorophyll cells.

### 2.4. Fractal Texture and Cuticular Microstructure of Avocado Peel During Ripening

The microstructural evolution of the avocado peel observed by CLSM (Figure 3a–l) showed changes consistent with ripening and senescence processes. The reduction in chlorophyll autofluorescence signal (Figure 3a,e,i) and its corresponding binarized quantification (Figure 3b,f,j) revealed a progressive decrease in the density of active photosynthetic cells associated with chlorophyll degradation and pheophytin accumulation. This behavior has been described as a marker of the transition to advanced stages of maturity and is related to the loss of antioxidant capacity and visual alteration of the peel [34,39].

Staining with Auramine O–Rhodamine B revealed alterations in cuticle integrity and thickness. Whereas UAP (Figure 3c) exhibited a continuous and compact cuticular layer, RAP (Figure 3g), and especially OAP (Figure 3k), showed progressive thinning, fragmentation, and irregularity of the cuticular contour. This transition reflects the lipid reorganization and degradation of cutin polymers and epicuticular waxes, processes that have been associated with the loss of cuticular barrier functionality during postharvest storage [48,49].

The binarization of the cuticular profiles (Figure 3d,h,l) confirmed this trend: the spatial complexity of the microreliefs and surface discontinuities was greater in UAP and progressively decreased in RAP and OAP. Quantitatively, significant differences (*p* < 0.05) were observed among all samples, with FDT values of 1.54 ± 0.02 for UAP, 1.51 ± 0.02 for RAP, and 1.47 ± 0.01 for OAP. Higher FDT values indicate more complex or rougher surfaces, consistent with the irregular morphology observed in the binarized contours of UAP (Figure 3d). The reduction in FDT reflects a simplification of peel surface topology, likely associated with cuticular degradation and the loss of structural integrity during ripening. Similar modifications have been reported in ripening fruit tissues, where changes in cuticle architecture and cell wall organization accompany physiological maturation and senescence processes [39,41,49]. Likewise, studies based on atomic force microscopy have shown decreases in surface roughness parameters (Ra and Rq) during the ripening of apples and bananas, supporting the structural simplification observed in the present study [50,51]. The microstructural parameters obtained using ESEM, CLSM, and image analysis revealed a strong association between tissue degradation, cuticular morphology, chlorophyll cell integrity, and the accumulation or extractability of phenolic compounds during ripening.

The microstructural parameters, together with the phenolic and antioxidant profiles presented in Table 1, show a clear inverse relationship, given that as structural integrity decreases, TPC and antioxidant capacity increase. OAP, which exhibited the lowest FDT and the highest degree of cell collapse, also showed the highest TPC and the highest ABTS•+ and FRAP values. These findings support the hypothesis that deterioration of the cuticle and cell structure improves the release, solubilization, and extractability of both free and bound phenolic compounds. A similar dependence of extractable phenolic compounds on tissue disorganization has been described in Hass avocado residues subjected to different extraction intensification strategies (ultrasound, enzymes, and optimized solvent systems), in which the disruption of cuticle–phenol–lipid interactions significantly increased the recovery and antioxidant capacity of peel extracts [52,53].

The results described above are consistent with recent biorefinery approaches and agro-industrial waste valorization studies, which describe avocado peel as a structurally complex matrix in which phenolic compounds, including procyanidins, quercetin derivatives, and hydroxycinnamic acids, are partially associated with indigestible polysaccharides and cell wall components. Mechanical or chemical disruption of the outer tissues not only improves extraction yields, but also enhances the interfacial and techno-functional properties of phenolic-rich extracts intended for food applications [28,54,55].

From a postharvest engineering perspective, these structural changes imply a progressive loss of mechanical strength and the ability of the peel to regulate mass transfer. The reduction in surface roughness and cuticle thinning decreases protection against water loss and microbial invasion, thereby compromising shelf life and sensory quality. Therefore, morphochemical characterization using CLSM and fractal texture analysis (FTA) is proposed as a valuable tool for modeling deterioration kinetics and optimizing storage strategies and edible coatings, with direct applications in postharvest conservation engineering for ‘Hass’ avocado and other high-value fruits.

### 2.5. Principal Component Analysis of Ripening-Associated Changes in Avocado Peel

Principal component analysis (PCA) was performed to integrate color attributes, fractal dimension texture (FDT), chlorophyll fluorescence (λ 450), total phenolic content (TPC), and antioxidant capacity (ABTS and FRAP) during avocado peel ripening. The first two principal components explained 99.0% of the total variance, with PC1 and PC2 accounting for 67.40% and 31.60%, respectively, indicating that the selected variables captured most of the physicochemical, structural, and biochemical changes associated with ripening.

The PCA analysis clearly separated unripe (UAP), ripe (RAP), and overripe (OAP) samples, reflecting the progressive transformation of avocado peel during ripening. UAP samples were positively associated with L*, Hue°, λ450, and FDT, suggesting greater chlorophyll retention, greener coloration, and higher structural integrity. The close relationship between FDT and chlorophyll fluorescence is consistent with previous reports describing the preservation of cuticular architecture and cellular organization in less mature tissues [40,41,49]. Likewise, changes in peel color are well-recognized indicators of avocado ripening and have been associated with chlorophyll degradation and metabolic changes occurring during postharvest storage [8,46].

In contrast, OAP samples were strongly associated with TPC, ABTS, and FRAP, indicating greater phenolic extractability and antioxidant activity at advanced ripening stages. Similar trends have been reported for avocado by-products, where peel tissues constitute an important reservoir of phenolic compounds with high antioxidant potential [25,28,55]. The increase in antioxidant activity may be related to the structural disassembly of peel tissues during ripening, facilitating the release and accessibility of bioactive compounds previously embedded within the cellular matrix.

FDT was located opposite to TPC and antioxidant variables in the PCA space, suggesting an inverse association between structural complexity and antioxidant potential. This behavior agrees with previous reports describing progressive modifications in cell wall organization, cuticular structure, and tissue morphology during fruit ripening and senescence [34,37,39]. Although PCA does not establish causality, the observed distribution suggests that structural degradation of the peel may occur simultaneously with enhanced phenolic extractability and antioxidant activity. RAP samples occupied an intermediate position between UAP and OAP, reflecting a transitional physiological stage characterized by partial chlorophyll degradation and moderate antioxidant activity. This intermediate clustering supports the concept that ripening is a continuous process involving coordinated structural, optical, and biochemical modifications rather than abrupt changes in a single attribute.

**Figure 4:**
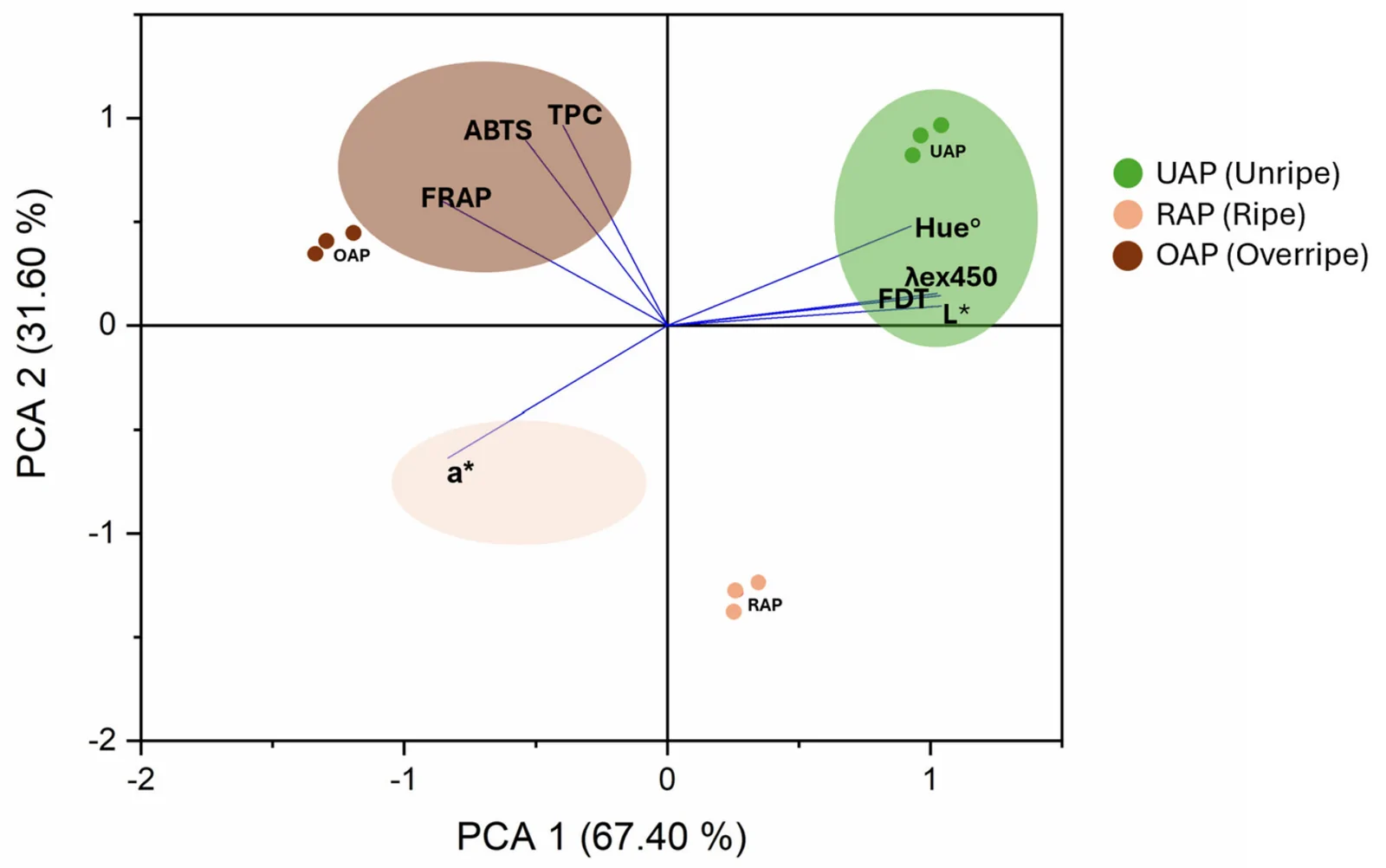
Principal component analysis (PCA) biplot showing the relationships among color parameters, chlorophyll fluorescence (λex = 450 nm), fractal dimension texture (FDT), total phenolic content (TPC), and antioxidant capacity during avocado peel ripening.

Overall, PCA revealed a strong association among microstructural integrity, chlorophyll fluorescence, color attributes, and antioxidant composition during avocado peel ripening. The substantial contribution of FDT to sample discrimination highlights its potential as a novel microstructural indicator of peel senescence and quality changes during ripening. Furthermore, the close association of OAP samples with phenolic compounds and antioxidant capacity reinforces the potential valorization of overripe avocado peel as a source of bioactive compounds for food applications [16,30] (Figure 4).

The results of this study demonstrate that avocado peel, especially at the overripe stage, has high potential as a source of bioactive compounds with strong antioxidant capacity, opening the possibility of utilizing this traditionally discarded by-product as a functional ingredient in food, cosmetic, and pharmaceutical formulations, in line with current biorefinery trends for agro-industrial waste valorization. The findings reveal a close relationship between microstructural degradation, pigment metabolism, and phenolic accumulation, highlighting the importance of integrating microscopy, fractal analysis, and biochemical data to understand how microstructure modulates phenolic availability and antioxidant behavior. This integrated approach provides valuable information for the development of postharvest quality assessment tools and new strategies for peel valorization in food engineering processes. Likewise, this work establishes a basis for comparative studies among maturity stages, cultivars, and production lines differing in cell wall metabolism and phytochemical accumulation. Nevertheless, further studies are required to clarify the enzymatic mechanisms responsible for structural modifications, the spatial localization of bioactive compounds within specific tissue layers, and the scalability of extraction processes. Future integration of microscopic techniques with vibrational spectroscopy (Raman and FTIR) and transcriptomic analyses will provide a deeper understanding of the interactions among structure, physiology, and phytochemical composition during fruit development and senescence.

## 3. Materials and Methods

### 3.1. Biological Material

Peels of ‘Hass’ avocados (*Persea americana* Mill.) at the unripe (UAP), ripe (RAP), an overripe (OAP) stages were collected from a local supermarket in Querétaro. A 20 kg batch was manually separated from the pulp, and the isolated peels were stored at −17 °C until analysis. For chemical and antioxidant analyses, a portion of the peels was dried in an oven at 75 °C for 5 h until reaching a moisture content of 2–3%, ground, and passed through a 60-mesh sieve to obtain particles smaller than 250 µm. The resulting powder was pulverized using a mechanical mill (IKA M20, Staufen im Breisgau, Germany). Samples intended for microstructural analyses were prepared separately from intact peel sections, as described in Section 3.5. The chemical composition was determined according to the AOAC methods [56], including ash (Method 923.03), protein (Method 954.01; N × 6.25), crude fat (Method 920.39), and dietary fiber according to Shiga et al. [57]. Total carbohydrate content was calculated by difference.

### 3.2. Color Measurement

Color was measured using the CIELAB system (MubuScan, Hunter-Lab, Reston, VA, USA). Results were reported as lightness (L*), chromatic coordinates (a* and b*), chroma (C*), and hue angle (Hue°). Values are expressed as the mean of 20 measurements. The L*, a*, and b* values were used to calculate chroma (C*) according to Equation (1) [58] and Hue° according to Equation (2) [59].

(1) $$C^{*}=\sqrt{a^{2}+b^{2}}$$

(2) $$Hue{}^{\circ}=arctg\;b^{*}/a^{*}$$

### 3.3. Total Phenolic Content (TPC)

Ethanolic extracts were obtained following the methodology described by Cardador-Martínez et al. [60]. Briefly, 1 g of avocado peel was mixed with 10 mL of 70% ethanol. The flasks were protected from light and maintained under constant agitation for 24 h. Subsequently, the samples were centrifuged at 5000 rpm for 10 min, and the precipitate and supernatant were collected separately and stored at 4 °C until analysis. Total phenolic content (TPC) was determined according to the method described by Sánchez-Quezada et al. [23]. TPC was quantified using a gallic acid calibration curve (0.1 mg/mL) and expressed as mg gallic acid equivalents (GAE)/g dry weight (d.w.).

### 3.4. Determination of Antioxidant Capacity by DPPH, ABTS•+, and FRAP

The DPPH radical scavenging assay was performed according to the method described by Sánchez-Quezada et al. [23] with minor modifications. Briefly, 20 μL of extract and 200 μL of DPPH• solution were added to each well of a 96-well microplate. The plate was incubated in the dark at room temperature for 30 min, and the absorbance was measured at 515 nm. The FRAP assay was performed by mixing 25 μL of extract with 175 μL of FRAP reagent in a microplate. The mixture was incubated at 37 °C for 15 min according to Torres-León et al. [61], and the absorbance was recorded at 593 nm. The ABTS•+ radical decolorization assay was carried out according to Re et al. [62]. The ABTS•+ radical cation was generated by reacting 7 mM ABTS with 2.45 mM potassium persulfate in distilled water and allowing the mixture to stand in the dark at room temperature for 16–17 h. Finally, absorbance was measured at 734 nm using a spectrophotometer (Multiskan Microplate Photometer, Thermo Scientific, Waltham, MA, USA). Antioxidant capacity was calculated using a Trolox standard curve and expressed as µmol Trolox equivalents (TE)/g dry weight (d.w.) of peel.

### 3.5. Histochemical Characterization

#### 3.5.1. Environmental Scanning Electron Microscopy (ESEM)

Avocado peel microstructure was observed using an environmental scanning electron microscope (ESEM; EVO LS10, Carl Zeiss, Jena, Germany). Approximately 1 × 1 cm sections of the isolated avocado peel were mounted on aluminum stubs using double-sided conductive carbon tape, and images were acquired with a secondary electron detector operating at 20 kV. Additionally, elemental microanalysis of the peel cross-section was performed using an energy-dispersive X-ray detector (EDX; Quantax 200, Bruker, Karlsruhe, Germany), following the methodology described by Pérez-Barcena et al. [63].

#### 3.5.2. Confocal Laser Scanning Microscopy (CLSM)

Confocal laser scanning microscopy (CLSM) enables the visualization of different tissue structures in combination with multicolor fluorescent staining, allowing their identification according to their chemical composition [64]. For the histological study, cross-sections (14 µm thick) were prepared using a cryostat (Leica CM1850, Nussloch, Germany). The sections were mounted on microscope slides and observed using a CLSM (LSM 710, Carl Zeiss, Germany). Samples were sequentially stained with Calcofluor White (0.1% aqueous solution, λex = 405 nm (Sigma-Aldrich, St. Louis, MO, USA; product no. 18909) to visualize cell wall polysaccharides (cellulose and hemicellulose), Rhodamine B (0.1% aqueous solution, λex = 514 nm; Sigma-Aldrich, St. Louis, MO, USA; product no. 72485) for carbohydrates, and Auramine O (0.1% aqueous solution, λex = 450 nm; Sigma-Aldrich, St. Louis, MO, USA; product no. 861030) to visualize the presence and distribution of the cuticle for 1 min [40,64]. Finally, the chlorophyll autofluorescence spectrum was obtained using the spectral channels method (650–700 nm) [65]. Images were acquired using a 20×/0.8 objective lens and stored in TIFF format with a resolution of 1024 × 1024 pixels. Fluorescence intensity was determined for each component and related to its concentration.

#### 3.5.3. Morphological Analysis Using Microscopy Techniques and IA

To study the surface and cross-sectional structure of the samples, images obtained by ESEM and CLSM were analyzed using ImageJ 1.52a software (National Institutes of Health, Bethesda, MD, USA). Image analysis was used to quantify the microstructural changes associated with ripening. For this purpose, 50 measurements were obtained for each tissue layer from ESEM and CLSM images using ImageJ 1.52a software. Original CLSM images were processed to generate binary images using the threshold tool (197/255). Area (A, µm^2^), perimeter (P, µm), Feret diameter (µm), aspect ratio (AR), and circularity were determined from 50 measurements [66]. To quantify the microstructural complexity of the peel surface, CLSM images stained with Auramine O and Rhodamine B were subjected to fractal analysis. The images were converted to 8-bit grayscale and binarized using a standardized threshold to isolate the cuticular contours. Fractal dimension texture (FDT) was calculated using the box-counting method with the FracLac 2015 plugin for ImageJ 1.52a software (National Institutes of Health, USA). Higher FDT values were interpreted as indicators of greater geometric complexity and surface roughness [43].

### 3.6. Statistical Analysis

Results are presented as mean ± standard deviation. Data were analyzed using one-way analysis of variance (ANOVA), and mean comparisons were performed using Tukey’s multiple comparison test at a significance level of *p* ≤ 0.05. Statistical analyses were conducted using MINITAB^®^ 16 software (Minitab Inc., State College, PA, USA). Principal component analysis (PCA) was performed using OriginPro 2018 (OriginLab Corporation, Northampton, MA, USA) to evaluate the relationships among color parameters (L*, a*, and Hue°), chlorophyll fluorescence (λex = 450 nm), fractal dimension texture (FDT), total phenolic content (TPC), and antioxidant capacity (ABTS and FRAP). PCA was conducted using a correlation matrix, and the first two principal components were retained for interpretation.

## 4. Conclusions

Hass avocado peel showed changes in chemical composition, color attributes, antioxidant properties, and microstructure across ripening stages. ESEM and CLSM revealed progressive changes in tissue organization and chlorophyll-containing structures, while FDT decreased from unripe to overripe peel, reflecting differences in surface structural complexity among ripening stages. Concurrently, overripe peel exhibited the highest total phenolic content and antioxidant capacity, suggesting that structural changes during ripening may favor the extractability of phenolic compounds. Principal component analysis further demonstrated a strong association between microstructural integrity, color attributes, and chlorophyll fluorescence, while phenolic content and antioxidant activity were associated with advanced ripening stages. The opposite positioning of FDT relative to TPC, ABTS, and FRAP suggests that structural disassembly of the peel occurs simultaneously with increased total phenolic content and antioxidant potential. These findings highlight FDT as a promising microstructural indicator of peel senescence and ripening progression. Overall, this study provides a comprehensive framework integrating microscopy, fractal analysis, fluorescence, and biochemical characterization to understand the structural and functional changes occurring in avocado peel during ripening. Furthermore, the high phenolic content and antioxidant capacity observed in overripe peel reinforce its potential as a valuable source of bioactive compounds for food, cosmetic, and pharmaceutical applications, contributing to the sustainable valorization of avocado processing by-products. Future studies should focus on the identification and quantification of individual phenolic compounds, elucidating the enzymatic and molecular mechanisms underlying peel structural disassembly, determining the spatial localization of bioactive compounds within specific tissue layers, and evaluating the scalability of extraction technologies for industrial applications. The integration of advanced spectroscopic and omics-based approaches may further improve our understanding of the interactions between microstructure, physiology, and phytochemical composition during fruit ripening and senescence.

## Figures and Tables

**Table 1:** Summary of the chemical composition and physicochemical properties of Hass avocado peel at different ripening stages.

**Chemical Composition (%)**			
Compound	UAP	RAP	OAP
Proteins	8.93 ± 0.43 ^a^	8.89 ± 0.09 ^a^	8.51 ± 0.09 ^a^
Lipids	10.47 ± 0.32 ^a^	10.52 ± 0.38 ^a^	9.67 ± 0.21 ^b^
Ash	3.39 ± 0.03 ^a^	3.51 ± 0.04 ^a^	3.41 ± 0.05 ^a^
Total carbohydrates	77.21 ± 0.23 ^b^	77.08 ± 0.50 ^b^	78.40 ± 0.21 ^a^
Total dietary fiber	61.33 ± 0.75 ^b^	66.89 ± 0.32 ^a^	66.62 ± 0.31 ^a^
**Physical Properties**			
L*	30.35 ± 0.41 ^a^	23.77 ± 1.69 ^b^	13.16 ± 1.10 ^c^
a*	−19.45 ± 0.38 ^a^	−0.19 ± 0.06 ^b^	1.98 ± 1.20 ^c^
b*	15.65 ± 1.76 ^a^	10.15 ± 2.20 ^b^	5.94 ± 1.20 ^c^
Hue°	141.27 ± 2.89 ^a^	91.15 ± 0.64 ^b^	72.52 ± 7.07 ^c^
C*	25.04 ± 1.37 ^a^	10.16 ± 2.20 ^b^	6.29 ± 1.49 ^b^
**Nutraceutical Composition**			
Total phenolic compounds (mg GAE/g)	153.58 ± 7.45 ^b^	87.28 ± 3.35 ^c^	168.02 ± 3.28 ^a^
DPPH (μM TE/g)	943.81 ± 5.40 ^a^	876.74 ± 6.18 ^c^	924.84 ± 8.70 ^b^
ABTS•+ (μM TE/g)	383.72 ± 4.62 ^b^	327.75 ± 7.09 ^c^	410.25 ± 1.01 ^a^
FRAP (μM TE/g)	621.44 ± 9.65 ^b^	548.53 ± 2.64 ^c^	782.84 ± 2.62 ^a^

The results are expressed as the mean ± SD of three independent experiments. Different superscripts represent statistical differences between rows by the Tukey test (*p* ≤ 0.05). Unripe avocado peel: UAP; ripe avocado peel: RAP; overripe avocado peel: OAP; Lightness, L*; green–red chromatic coordinate, a*; blue–yellow chromatic coordinate, b*; chroma, C*; Hue angle, Hue°; mg GAE/g: milligrams of gallic acid equivalents per g of dry peel; µM TE/g: micromole of Trolox equivalents per g of dry peel.

**Table 2:** Morphological features and fluorescence intensity of chlorophyll cells.

Chemical Composition (%)			
Compound	UAP	RAP	OAP
A (µm^2^)	4.47 ± 6.02 ^a^	2.57 ± 3.56 ^b^	1.38 ± 0.73 ^c^
P (µm)	7.35 ± 6.1 ^a^	5.49 ± 4.71 ^b^	4.06 ± 0.75 ^c^
Feret diameter (µm)	106.96 ± 47.59 ^b^	112.24 ± 42.08 ^a^	72.65 ± 50.60 ^c^
AR	1.97 ± 0.89 ^a^	1.76 ± 0.75 ^b^	1.18 ± 0.26 ^c^
Circularity	0.78 ± 0.21 ^c^	0.82 ± 0.21 ^b^	0.93 ± 0.09 ^a^
λex = 450 nm	6.16 ± 0.80 ^a^	4.44 ± 0.66 ^b^	0.48 ± 0.11 ^c^

The results are expressed as the mean ± SD of three independent experiments. Different superscripts represent statistical differences between rows by the Tukey test (*p* ≤ 0.05). Unripe avocado peel: UAP; ripe avocado peel: RAP; overripe avocado peel: OAP; A: area, P: perimeter; AR: aspect ratio; λex: fluorescence excitation.

## Data Availability

The original contributions presented in this study are included in the article. Further inquiries can be directed to the corresponding authors.

## Supplementary Materials

The following supporting information can be downloaded at: https://www.mdpi.com/article/10.3390/molecules31183280/s1, Figure S1: Avocados at different ripeness states and CIELAB color parameters.

## Abbreviations

The following abbreviations are used in this manuscript:

UAP	Unripe avocado peel
RAP	Ripe avocado peel
OAP	Overripe avocado peel
L*	Lightness
a*	Red–green chromatic coordinate
b*	Yellow–blue chromatic coordinate
C*	Chroma
Hue°	Hue angle
TPC	Total phenolic compounds
DPPH	2,2-Diphenyl-1-picrylhydrazyl
ABTS•+	2,2′-Azino-bis (3-ethylbenzothiazoline-6-sulfonic acid) radical cation
FRAP	Ferric reducing antioxidant power
CW	Cell wall
OW	Outer wax layer
IW	Inner wax layer
EP	Epidermis
Hy	Hypodermis
PP	Pericarp parenchyma
ESEM	Environmental scanning electron microscopy
A	Area
P	Perimeter
AR	Aspect ratio
λex	Fluorescence excitation wavelength
FDT	Fractal dimension texture
