# Supplementary material for: Changes in Phenolic Compounds, Antioxidant Capacity, Color, and Microstructure of Hass Avocado (Persea americana) Peel During Ripening
Source: Molecules. 2026 Sep 16;31(18):3280. doi: 10.3390/molecules31183280 (PMC13609036; doi:10.3390/molecules31183280)
Supplement: Supplementary file 1 [file molecules-31-03280-s001.zip › molecules-4525184-supplementary.pdf]

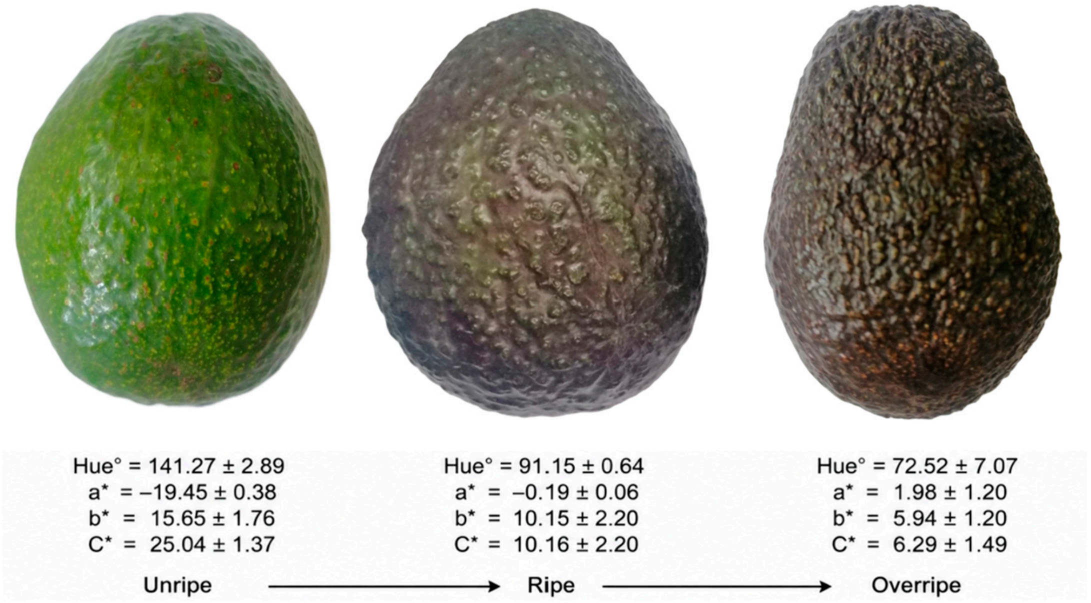

Figure S1. Avocados at different ripeness states and CIELAB color parameters.
